# Stress coping strategies among medical students and trainees in Saudi Arabia: a qualitative study

**DOI:** 10.1186/s12909-020-02039-y

**Published:** 2020-04-22

**Authors:** Noura Abouammoh, Farhana Irfan, Eiad AlFaris

**Affiliations:** King Saud University Chair of Medical Education Research and Development, Department of Family and Community Medicine, College of Medicine, King Saud University, Riyadh, Saudi Arabia

**Keywords:** Coping strategies, Medical students, Medical interns, Qualitative

## Abstract

**Background:**

Stress is very common among medical students and trainees. Chronic stress has multiple negative mental and physical health consequences. This study explores medical students and interns’ stressors and their coping strategies.

**Methods:**

This is a qualitative study involving four focus group discussions with final year medical students (8 males, 10 females) and medical interns (8 males, 7 females). The study was conducted between October 2017 and January 2018 in the premises of a large medical school in Riyadh. After verbatim transcription, framework thematic analysis of the data was performed using NVivo software.

**Results:**

Promoting the reward feeling of the medical profession was seen as an active stress-coping strategy among medical students. Planning and time management was seen by some participants as stressful while others felt it was a stress-coping strategy. The perception of stress among medical students was seen as a normal feeling. Avoiding discussions on medical matters, building relationships with the other sex and smoking were reported as avoidant stress-coping strategies.

**Conclusion:**

Medical students and interns are **still** struggling to achieve healthy stress-coping strategies. There is a great need for stress management programs to help fostering the students’ coping skills.

## Background

Stress is generally a situation where the demands exceed the capacity of an individual to respond and can potentially have negative physical and psychological consequences [1, 2]. It is generally a combination of two elements; the inability to meet demands placed or the judgment made by the individual of being unable to meet the demands [3]. Stress is prevalent throughout the field of medicine at both undergraduate and postgraduate levels and is a common feature of medical students’ lives [4]. According to the theory of mental toughness, experiencing some reasonable stressors, with periods of recovery in between can make an individual mentally and physically stronger [5]. However, chronic stress is associated with increased chances of depression, relationship difficulties, anxiety and suicide [6].

The medical school is a time of significant psychological distress for medical students as they face numerous academic challenges that make them more prone to stress and anxiety when compared to students of other disciplines [7]. The most important stressors experienced during studying and training in the medical school include high self and external expectations, stressful learning environment, with heavy academic tasks and high required academic grades [8]. The effect caused by theses stressors include the state of students’ well-being and their success, in the long term [9, 10].

During the medical school years, students utilize various adaptive coping strategies to manage stress [4, 11] and to cope with the external and internal demands [2]. Medical students believe that their lives would be improved if these challenges are removed [4]. However, stress will continue even in the transition period from being a student to becoming an intern, where they bear the added responsibility of their patients’ care [10].

Coping theory is defined as “*constantly changing cognitive and behavioral efforts to manage specific external and internal demands that are appraised as taxing or exceeding the resources of the person”* [11]*.* Coping theory is classified into two independent parameters namely; focus-oriented theories and approach-oriented theories. While the first recognizes peoples’ internal resources and mental capacities for evaluating how competently they can adapt to a situation, the latter concerns with how concrete the coping mechanisms are [12]. One of the most frequently used focus-oriented approaches was provided by Ebata and Moos. Active (positive or functional) and avoidant (negative or dysfunctional) coping approaches were defined based on whether a person’s response is directed towards the stressor or away from it [13]. Coping is dependent on personality and perceptions about life experiences and the strategies adapted can differ by individuals. However, overall, the main aim is reducing stress and reaching a balanced state of functioning.

The information available on stress and coping strategies suggests that coping mechanisms differ between the two sexes. Women adopt more emotion-focused approach and are more likely to resort to negative avoidant coping strategies, than men [14, 15].

In view of the importance of this topic there is a need to develop better understanding of the different stressors experienced by medical students and their coping mechanisms. Although, many studies on stress and coping strategies were conducted, the literature is still short in qualitative research to explore students’ experiences with stress from their perspectives [15]. The aim of the current study is to explore the coping strategies used by medical students and interns to cope with different stressors.

## Methods

### Study design and participants

This phenomenological study involved medical students and interns at the college of medicine, King Saud University (KSU), Riyadh. Focus group discussion method was employed to explore their stress coping strategies.

### Setting and curricular framework

Students must have completed a six years medical school program before they are enrolled to a one-year mandatory internship to get their Bachelor of Medicine and Surgery. The first year in medical school is preparatory during which they study chemistry, physics and English language. The following two years start with system-oriented basic medical science courses, followed by one year of preclinical courses and two years of clinical clerkship. During the last year, students rotate between medicine, surgery and pediatric specialties with a main focus on practical sessions. Evaluation, during the last year, include theoretical and practical exams to be held after each rotation. This is followed by a mandatory internship year comprising two months clinical rotation in each of medicine, surgery, obstetrics and gynecology, pediatrics in addition to two elective clinical rotations. Although interns are expected to have some responsibilities in terms of patients’ care, they are not required to undertake written or work place-based assessments. The internship training is structured only in a few departments and is less structured in others. Interns are eligible for the Saudi Medical Licensing Exam (SMLE) that is held twice a year.

### Sampling and recruitment

Participants were recruited to four focus group discussions during the academic year 2017/2018. Each group was formed of 7–10 participants. Two focus groups were from fifth-year students and two were interns. Following the local cultural norm in Saudi Arabia, male and female focus groups were kept separately.

Leaders from each batch were contacted personally by one of the researchers (EA) and were requested to recruit ten students of the same batch to join a focus group discussion. The researchers shared an information sheet explaining the aim and objectives of the study to the leaders of each group who were requested to distribute copies of the sheet to their colleagues when inviting them to join the study. Participation was voluntary. The principal investigator (NA), in the presence of another member of the research team (FI), conducted the focus group discussions. Both researchers are faculty in the College of Medicine with special interest in medical education. Refreshments were served during the focus group discussions to keep it informal and relaxing for the participants.

### Data collection

Data was collected between October 2017 and January 2018. Before each focus group discussion, demographic information sheets were filled out by the participants. This included information such as educational level, sex and GPA. Focus group discussions were conducted by NA (MD, PhD) who is a qualitative researcher and FI (MD FRCGP, MCPS). Both were females who are responsible for the teaching of female medical students. The discussions took place in a seminar room in the College of Medicine in the presence of the two interviewers and participants only. The discussions were audio-recorded and lasted between 45 and 80 min. Participants were given the choice to speak Arabic (the native language of all participants) or English. The discussions were led by a topic guide that was developed based on the aims of the current study and previous similar studies [16–18].

Flexibility was observed when following the structured topic guide to allow the emergence of new themes, considering the interactive nature of the focus group.

The discussion started with a general question “How does your life look like as medical students/interns?”. This was followed by a discussion involving stress coping strategies and support system. To ensure covering all key areas raised by the participants, each focus group discussion was analyzed before starting the next group discussion.

### Data analysis

Focus group recordings were transcribed professionally by external transcribers. To preserve the meaning, transcripts were analyzed in their original language and only quotes were translated into English.

Two coders (NA and FI) were involved in coding the data. The process of data analysis started with refamiliarization with the data. Codes of the first focus group were developed independently by the two authors who undertook the description of the content. The developed codes were similar and were discussed before the agreement on a coding frame was established. These were revisited after the second, third and fourth focus group discussions with more details of the pattern across the discussions. Themes were developed based on the identified pattern of the codes. Coding was completed using emergent themes which were investigated until saturation was achieved. Inductive thematic analysis was employed. The themes were derived from the collected data and the topic guide was partially developed based on previous similar studies. NVivo software version 11.4.2 was used to manage the data.

## Results

Eight male and ten female students in addition to eight male and seven female medical interns were interviewed in four different focus group discussions (Table 1).

**Table 1:** Participants’ demographics

Male participants number	Level (age)	GPA/5	Female participant number	Level (age)	GPA/5
1	Fifth year (23)	4.72	1	Fifth year (24)	3.75
2	Fifth year (23)	4.5	2	Fifth year (23)	4.02
3	Fifth year (23)	4.47	3	Fifth year (23)	4.71
4	Fifth year (23)	4.25	4	Fifth year (22)	4.21
5	Fifth year (22)	4.85	5	Fifth year (12)	3.31
6	Fifth year (23)	4.7	6	Fifth year (23)	4.3
7	Fifth year (23)	4.6	7	Fifth year (23)	4.44
8	Fifth year (22)	4.6	8	Fifth year (23)	3.5
9	Intern (24)	4.5	9	Fifth year (23)	4.4
10	Intern (24)	4.5	10	Fifth year (23)	3.4
11	Intern (23)	4.2	11	Intern (24)	4.6
12	Intern (25)	4.5	12	Intern (23)	4.3
13	Intern (24)	4.3	13	Intern (23)	4
14	Intern (25)	4.3	14	Intern (24)	4.7
15	Intern (25)	4.4	15	Intern (25)	4.2
16	Intern (24)	4	16	Intern (24)	4.7
			17	Intern (24)	4.6

It was observed by the two interviewers that the participants were comfortably expressing their views and experiences and were unlikely to have been discouraged to speak freely. Some participants adopted active coping strategies while others adopted avoidant strategies (Table 2). Examples of active coping strategies included cognitive self-rewarding, planning and time management, and socialising and communicating. On the other hand, examples of avoidant strategies were avoiding medical discussions with friends, building relationships with the other sex, smoking and physical exercise. The following themes emerged from the focus group discussions:

A) Active strategies

**Table 2:** Process of developing themes from focus group discussions with medical students and interns

Primary Codes	Categories	Organizing Themes
- View about future social and financial status to self-motivate - instant treats to self-motivate - Reframe stress to be inevitable and healthy - Organizing short-term plans - Relationship of marital status of students to their time management ability - Socializing - Physical exercise, reading and volunteering	1- Cognitive self-rewarding 2- Planning and time management 3- Socializing and communication 4- Engaging in recreational activities	Active strategies
- Refraining from medical related talks with friends - Refraining from medical talks with overachievers - Building relationships with the other gender as self-distraction or for ventilation - Smoking to relief stress - Smoking as self-distraction	1- Avoiding medical discussions 2- Building relationships with the other sex 3- Smoking 4- Physical exercise	Avoidant strategies

### Cognitive self-rewarding

Participants felt that doctors are respected by the society. The medical profession was viewed by male participants as prestigious and this is an emotional reward for them. This self-satisfaction reward compensates for the stress they feel throughout the medical school years. For example, a participant noted:

*“We have a stressful life, but I think we are greatly satisfied. Sometime I compare myself to my relatives and I believe that I have a better social status, everybody is praising medical students. You are a doctor, you have something ... So, I am stressed, but I’m doing good” (Male1, student)*

Male participants believed that stress is an inherent part of life that is inevitable and is lifelong. If a person wants a better social and financial life, it should be handled and dealt with in a healthy way.

*“Consultants have good lives, they’re used to stressful situations all the time, so they are okay with it” (Male14, intern)*

Another example:

*“For myself, I realized that I have to live with this stress, because I think having the stress at work or during studies, is healthier. Otherwise, I’m not going to survive it” (Male1, student)*

### Planning and time management

Students came up with different views regarding planning and time management during the focus group discussions. Some felt that practising a self-created pre-set schedule to manage time was a good way to reduce stress before the exams.

For example:

*“I used to create a time table … like a schedule for every hour what I should study. I organize myself. That’s what relieves my stress, because I see that I will finish well before the exams” (Male7, student)*

Other students, however, had an opposite view, as shown by the following comment from an intern:

*“No, I cannot follow a self-set schedule. It’s like having someone nagging on your head and that makes me more stressed. Besides, what if I couldn’t follow that schedule? I’ll be living in constant guilt and stress all at once!” (Female15, intern)*

Female participants observed that their married colleagues managed their time and stress effectively. A single female student noted:

*“you see them [married students] always taking advantage of their free time in the college by studying and finishing something in the classes alone while we are hanging because they have responsibilities at home” (Female1, student)*

Another single female student added:

*“Married students may be busier than us, but they have a clearer vision of what they want from life so their plan to achieve what they want with the minimum amount of stress seems doable.” (Female8, student)*

Nonetheless, the only married participant reported:

*“I have a four-year-old son, so my journey will never be as easy as it is with my friends. In addition to taking care of myself and my studies, I have to take care of a house, a son and a husband. It’s overwhelming sometimes” (Female15, intern)*

### Socializing and communication

To lessen the stress, students spend time with family and attend gatherings, where no one speaks about medicine.

*“If I am stressed I go and sit with my family, I don’t show that I am stressed. They usually ask whether the studies are going well. I assure them and change the topic, this is how I relieve my stress. (Female4, student)*

Unlike their male counterparts, female participants resort to instant and materialistic self-rewards which were seen as pleasurable experiences that gave them a feeling of happiness. This helped counterbalancing the negative feelings of stress. For example:

*“I literally reward myself when I finish a number of chapters or do well in the exams. I even may treat myself with ice-cream or throw a party to celebrate the achievement … we look forward to that” (Female5, student)*

### Engaging in recreational activities

Most participants accepted that engaging in recreational activities or hobbies such as, reading, physical exercise or voluntarily work helps to relieve stress and feel relaxed. Others believed that it’s a way of self-distraction rather than stress-relieving per se. For example, they may accuse themselves with “*wasting their time*” and may feel guilty after spending time doing what they like to do. One intern noted:

*“I started going to the gym … to control the stress … most of the time I feel guilty after going to the gym because I could spend my time studying for the exams or working on research” (Male9, intern)*

Other participants believed that they were not able to pursue their hobbies because of their busy schedules or as a way to avoid the guilt they may feel afterwards.

B) Avoidant strategies

### Avoiding medical discussions

Male participants mentioned that in order to reduce stress they may resort to non-medical friends or colleagues who refrain from medical and study-related discussions. This was believed to be common among medical students but hard to maintain.

*“Actually, I really hate to hang out with some medical students, because I know exactly what we’re going to discuss, it is all about studying, exams, professors... Even if we say: Do not discuss medical stuff! we eventually do” (Male4, student)*

On the other hand, the latter issue perceived among female participants as a way to control their own stresses rather than a self-distraction per se. One female participant explained that she doesn’t like to accompany some students as she believed that stress is a contagious feeling:

*“Some people are all the time stressed to an extent that you do not want to hear them talking and you cannot talk to them. I know what I am capable of and I do not want to be surrounded by negative people” (Female8, student)*

### Building relationships with the other sex

Most male participants believed that most students tend to have relationships with the other sex because they claim this releases their stress.

*“Being in an emotional relationship is something to distract you from medicine and the stresses of medicine” (Male4, student)*

He further explained:

*“It’s just talking to the other sex that gives you relief from or a way out of stress. They do not have to be from the same profession or anything. Because being emotionally stable, I think helps … it is kind of getting support” (Male4, student)*

When asked about the success of building relationships with the other sex in relieving stress, participants noted the following:

*“I think it’s not helping at all. It will not relieve stress nor make them study more, but they have to do it. It’s a distraction. Kind of trying something new, whatever that is” (Male5, student)*

Female students, however, believed that talking to male colleagues is more than a distraction and helps to relieve their stress.

*“I mean that stress will be relieved because he is more understanding. If I go and talk to a girl she might be thinking that I am incapable of doing things, girls usually judge you … I am just trying to be open in this issue” (Female10, student)*

On the other hand, a number of participants from both sexes considered these relationships as *“waste of time”* and *“religiously not acceptable”*.

### Smoking

Participants acknowledged that smoking is not an effective strategy to relieve stress; however, they do smoke when they are stressed. One participant explained the smoking phenomenon as:

*“One of the ways to escape stress is to try other new things in life” (Male7, student)*

Another participant explained:

*“If I’m under stress or anybody is under stress, will not start smoking, but if he is a previous or a frequent smoker, and he is under stress, he will start or smoke more” (Male8, student)*

All participants admitted that they have never been offered a cigarette by their friends and colleagues,

*“Even those who do smoke do not offer a cigarette to relieve your stress … Even if we ask, they would say no. Most of them know that this is wrong” (Male10, intern)*

A female participant explained:

*“I have noticed that after the second or third year of studying, especially in the medical field, girls in the university dormitory start to smoke cigarettes or water pipe but I do not see that it relieves their stress. They believe that the stress they are going through has forced them to smoke” (Female10, student)*

One male intern thinks that facing seriously stressful situations like that in the academia, in a young age may lead to taking “*impulsive decisions*” or “*irrational decisions*” to deal with stress; and smoking is one of them.

In general, participants initially started smoking as a pleasurable activity that helped in escaping stress, but later they perceive it as an additional emotional burden that needs to be dealt with. One participant mentioned:

*“Now it [smoking] is not a pleasure it’s a burden” (Male6, intern)*

#### Physical exercise

Although some participants engage in physical exercise as a way to relieve their stress, few mentioned that they engage in exercise to escape from studying. For example, one participant, who had the lowest GPA among the group, mentioned that she took physical exercise as a way to escape from studying. She explained:

*“I have been overweight since I was born however, two years ago, I decided to take an action. In my mind, I know that has nothing to do with improving my health, but rather to avoid studying” (Female5, student)*

## Discussion

The current study shows that the participants used more active coping strategies than avoidant strategies to cope with stress, though a significant proportion of them used avoidant coping strategies to escape from stress. This goes in line with other studies conducted in Nepal, Malaysia and Pakistan [19–21]. It was clear that male and female students differed in their stress coping strategies.

While avoidant strategies are associated with negative health outcomes, positive reframing is associated with improved well-being [22, 23]. Students and interns in this study have cognitively reframed stress as an expected and normal challenge. They accepted the fact that experiencing stress during medical school will help them to achieve socially and financially stable life in the future. On the other hand, the same students and interns practiced some avoidant strategies such as, avoiding medical-related discussions with friends and family members and engaging in activities to be distracted from the stressful environment.

It is clear that with all their attempts to positively reframe stress, participants are still trying to find ways to avoid it. However, the feeling of guilt after escaping from stress follows. This unavoidable outcome could add to the negative feeling they are already experiencing from stress.

Self-distraction was identified in the current study as a common avoidant coping strategy among medical students. This corresponds with many previous reported findings in medical students [19–21, 23]. The present study added that avoiding medical discussions with friends is a strategy that lessens stress by self-distraction; however, this strategy seems to prove its ineffectiveness as students inevitably bring up medically related conversations. Female participants, on the other hand, adopted the same strategy to cope with stress by avoiding dialogues with over stressed students or achievers rather than self-distraction per se.

Building relationships with the other sex was one of the self-distraction strategies which was not fully explored in the literature. This behaviour does not conform with the local culture in Saudi Arabia. However, it was practiced among participants and viewed by them as an avoidant coping strategy to stress. This issue was raised by the first group and discussed in the following focus groups, who recognised it as a self-distraction mechanism to cope with stress. Female participants, thought that this strategy can lessen stress due to their perception of married students as emotionally stable and more capable of managing their time properly.

As to out of class discussions with friends and others, male participants tend to avoid medical discussions with the other sex, while female participants take this opportunity to complain and ventilate their stress as they perceive their male counterparts as better listeners and less judgmental than female colleagues. The current study also added that participants acknowledge the ineffectiveness of this behaviour in relieving stress and this was taken to indicate that students need a safe setting and a protected and supportive environment to be able to express their feelings.

Although few participants used physical exercise as an avoidant strategy to escape stress, engaging in exercise or other hobbies was not generally considered by most participants as self-distraction. In contrast, this was also viewed as a way to relief stress. This might be due to the positive self-focused health benefits of exercise that they experienced which is not the case when spending time with friends or building relationships with the other sex.

Previous reports from Saudi Arabia [24, 25] identified smoking among medical students as yet another stress avoidant strategy. In the current exploratory study, smoking was perceived by male students as a kind of avoidant strategy but cannot be relied on to relieve stress. Most male students and interns tend to get back to smoking if they were ex-smokers. Female students may start smoking as a result of stress. In both cases, students and interns acknowledged the ineffectiveness of smoking in relieving their stress. Contrary to the findings of a previous study [24], students were not encouraged to smoke by their smoking colleagues.

Almost all participants of the present study adopted active coping strategies such as planning and acceptance rather than avoidant strategies such as denial, alcohol, and drugs and behavioral disengagement.

In line with previously published studies [14, 26], the findings of the present study showed that male and female participants differed in viewing stress and coping with it. Cognitive self-rewarding was identified as a common theme among both sexes. While, emotional rewards such as self-satisfaction with the expected future social and financial status were common among male students, instant self-rewards seemed to dominate female students thinking.

Adopting avoidant coping strategies is common among medical students. Previous studies found that avoidant stress-coping strategies were associated with psychiatric illness, shorter sleep duration, anxiety and depression [27–29]. Moreover, chronic stress was found to be associated with chronic fatigue, substance abuse and suicidal ideations among physicians [30]. While most published studies have highlighted the most frequently adopted stress-coping strategies among medical students, to our knowledge, the present study is the first to explore these strategies, their meanings and effectiveness from the perspectives of medical students and interns.

Generally, it appears from the findings of this study that medical students and interns were trying to find ways to lessen the negative effect of the stress they experience, in order to improve their quality of life, health and performance.

The students who participated in the present study may have avoided acknowledging or detailing their responses to the questions raised due to the sensitivity of the subject. We believe that more research needs to be done to allow better understanding of the nature of the relationships between the two sexes; and smoking among medical students in this conservative community. Similar research needs to be undertaken in the future at the international level to better understand the relationship between stress and relationships between both sexes among medical students. Furthermore, assessing students’ needs and exploring the best ways to support them in managing stress from their perspectives is highly desirable.

### Limitations

Male students may have felt less comfortable in reporting certain experiences because the interviewers were females. Theoretically, it could be speculated that the male students may not be as forthcoming in a focus group led by female interviewers. The Saudi community is conservative with separate teaching settings for both sexes. None the less, the interviewers felt that the participants were not inhibited when expressing their views and were willing to share details about their experiences.

## Conclusion

It is necessary not only to consider medical students’ education and professional training but also their quality of life during and after medical school years. There is a great need for medical schools to offer guidance and support to their students and to conduct stress management programs to help strengthening their coping skills. These programs could be designed based on the students’ needs.

## Data Availability

The datasets used and/or analysed during the current study available from the corresponding author upon request.

## Abbreviations

KSU: King Saud University
GPA: Grade Point Average
NVivo: Navigating viewpoints, images and value observed
MD: Medical Doctor
PhD: Doctor of Philosophy
FRCGP: Membership of the Royal College of General Practitioners
MCPS: Member of College of Physicians & Surgeons Pakistan
